# Recovery of Phenolic Compounds From *Elsholtzia ciliata* Leaves by Ultrasound‐Assisted Extraction and Their Antioxidant Application in Food

**DOI:** 10.1002/fsn3.72080

**Published:** 2026-07-06

**Authors:** Nguyen Phuong Vi Truong, Tran Diem Ai Chau, Huu Hieu Nguyen, Thi Anh Dao Dong

**Affiliations:** ^1^ Department of Food Technology, Faculty of Chemical Engineering Ho Chi Minh City University of Technology (HCMUT), VNU‐HCM Ho Chi Minh City Vietnam; ^2^ Department of Food Technology Thu Dau Mot University Ho Chi Minh City Vietnam

**Keywords:** chlorogenic acid, *Elsholtzia ciliata*, lipid oxidation, phenolic compound, ultrasound‐assisted extraction

## Abstract

*Elsholtzia ciliata* is a potential source of potent antioxidant phenolic compounds. This study investigated the ultrasound‐assisted extraction (UAE) of phenolic compounds (TPC) and chlorogenic acid (CGA) from *E. ciliata* (ELC) leaf extract. The study also evaluated the antioxidant efficacy of the extract during the preservation of 8F fat‐containing gummy candies. Using the response surface method (RSM), the optimal extraction temperature, time, and sonication time were predicted to be 77°C, 67 min, and 3.6 min, respectively. At the same time, ethanol concentration (60%), pH (5.5), and material‐to‐solvent ratio (1:18) were fixed based on preliminary selection. Under these conditions, TPC, CGA, and antioxidant activity reached 28.45 ± 0.5 mg gallic acid equivalents (GAE)/g dry weight (DW), 6.07 ± 0.18 mg/g DW, and 304.72 ± 0.83 μmol Trolox equivalents (TE)/g DW, respectively. Scanning electron microscopy (SEM) images showed that UAE conditions were favorable for the release of phenolic compounds. When incorporated into gummy candies, lipid oxidation was monitored over 49 days by measuring peroxide (PV) and thiobarbituric acid reactive substances (TBARS) values and compared to butylhydroxytoluene (BHT) and vitamin E. These results support the use of the ELC extract as a natural antioxidant to stabilize fatty foods.

## Graphical abstract

Ultrasound‐assisted extraction, optimized by response surface methodology, efficiently recovered antioxidant phenolics from *Elsholtzia ciliata* leaves at 77°C for 67 min with 3.6 min of sonication, using 60% ethanol, pH 5.5, and a 1:18 material‐to‐solvent ratio. The optimized extract contained 28.45 ± 0.50 mg GAE/g DW total phenolic content and 6.07 ± 0.18 mg/g DW chlorogenic acid, with antioxidant activity of 304.72 ± 0.83 μmol TE/g DW. Its efficacy in 8% fat gummy candies was evaluated against BHT and vitamin E by monitoring PV and TBARS over 49 days, supporting its potential as a natural antioxidant for lipid‐containing foods.

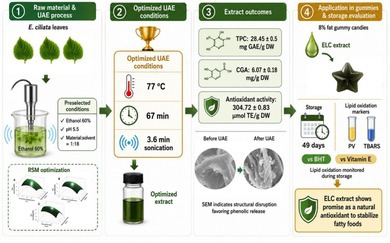

**Abbreviations**
AOAC: Association of official analytical chemists
BHT: Butylhydroxytoluene
CCD: Central composite design
CGA: Chlorogenic acid
Coeff. SC: Coefficient Standardized Coefficient
DPPH: 2,2‐diphenyl‐1‐picrylhydrazyl
DW: Dry weight
ELC:
*Elsholtzia ciliata* leaf extract
GAE: Gallic acid equivalents
HPLC: High‐Performance Liquid Chromatography
MDA: Malondialdehyde
PBD: Plackett–Burman design
PV: Peroxide value
RSA: Radical scavenging activity
RSM: Response surface methodology
SEM: Scanning electron microscopy
TBARS: Thiobarbituric acid reactive substances
TCA: Trichloroacetic acid
TE: Trolox equivalents
TMP: 1,1,3,3‐tetramethoxypropane
TPC: Total phenolic compounds
UAE: Ultrasonic‐assisted extraction

## 1 Introduction

Natural compounds, including phenolic compounds such as chlorogenic acid (CGA), are widely recognized for their potent antioxidant properties and health benefits, such as anti‐inflammatory and antimicrobial effects (Yusoff et al. 2022). *Elsholtzia ciliata* belongs to the genus *Elsholtzia*, which is part of the family Lamiaceae. ELC is a popular aromatic herb used in traditional medicine and culinary practices across Asia. Its leaves have been reported to contain considerable amounts of phenolic compounds, including phenolic acids and flavonoids (Pudziuvelyte et al. 2017, 2020), making it a promising source of natural antioxidants.

Ultrasound‐assisted extraction (UAE) has attracted increasing attention as a green and efficient technique for the recovery of phenolic compounds from plant materials. By disrupting cell walls and enhancing solvent penetration, UAE can improve the release of bioactive constituents. However, extraction efficiency strongly depends on operating parameters such as ultrasound intensity, frequency, temperature, and extraction time, which must be carefully optimized to prevent compound degradation (Bouafia et al. 2021; Lohani and Muthukumarappan 2021; Sim et al. 2019; Wang et al. 2024).

Previous studies have demonstrated the successful application of UAE in recovering CGA and phenolic compounds from many plants, including *Morus alba* (Martín‐García et al. 2022) and tobacco waste (Zeng et al. 2022). In addition, Pudziuvelyte et al. (2020) analyzed the phenolic contents in different parts of *E. ciliata* and also noted that UAE provided better compound recovery than traditional extraction (Pudziuvelyte et al. 2020). However, previous studies on ELC have mainly focused on phenolic compounds or on comparisons of extraction methods, with limited attention to optimizing UAE conditions to maximize the recovery of antioxidant compounds such as TPC and CGA (Pudziuvelyte et al. 2018). More importantly, the functional antioxidant performance of ELC extracts in real food matrices has not been clearly established. In addition, the use of ELC leaf extract as a natural antioxidant in gummy candy and its effectiveness in controlling lipid oxidation during storage have not yet been reported.

To the best of our knowledge, no previous study has integrated the optimization of UAE for ELC leaves with the evaluation of the resulting extract as a natural antioxidant in gummy candy during storage. Therefore, the present study aimed to optimize UAE conditions for maximizing TPC recovery from ELC leaves and to evaluate the antioxidant effectiveness of the extract in gummy candy during 49 days of storage. Antioxidant activity was assessed using the DPPH assay, while oxidative stability was evaluated through peroxide value and thiobarbituric acid reactive substances. This approach provides a more comprehensive assessment of *E. ciliata* extract for its potential use as a natural antioxidant in functional confectionery and broader food applications.

## 2 Materials and Methods

### 2.1 Plant Materials

ELC leaves were collected in October 2024 from Lam Dong (Vietnam). After being cleaned of dust, the leaves were dried in a heat pump dryer for 18 h at 42°C until they had 12%–13% moisture. The protein and ash contents were determined following the National Renewable Energy Laboratory's standard laboratory analytical procedure (Hames et al. 2008; Sluiter et al. 2008). Carbohydrate content was analyzed according to AOAC method 985.29 (Casterline et al. 1999). Lipid content was measured using AOAC method 996.06. Crude fiber was quantified based on AOAC method 962.09.

### 2.2 Chemicals and Reagents

Gallic acid, chlorogenic acid, and sodium carbonate were obtained from Sigma‐Aldrich (USA). HPLC‐grade solvents, including ethanol, methanol, and acetonitrile, were supplied by Fisher (USA). Additional reagents such as Folin–Ciocalteu's phenol, 2,2‐diphenyl‐1‐picrylhydrazyl (DPPH), and Trolox were purchased from Sigma‐Aldrich (USA).

### 2.3 Methods

#### 2.3.1 Ultrasound‐Assisted Extraction (UAE) Method

The extraction procedure was developed based on the UAE approach reported by Pudziuvelyte et al. (2018), with minor modifications to the solvent composition and extraction sequence. The bioactive compounds were extracted from ELC leaves using an ultrasound‐assisted method in an ultrasonic bath (Model: VC505, 500 W, 20 kHz, 230 V, Sonics, USA). Briefly, the effects of ultrasonic amplitude, sonication time, pH, extraction temperature, and extraction time on phenolic recovery were evaluated through single‐factor experiments.

Dried ELC leaf powder was suspended in distilled water. The suspension was sonicated for 1–9 min at an ultrasonic amplitude of 30%–90%. After sonication, ethanol was added to adjust the solvent to 60% (v/v) ethanol and to obtain a material‐to‐solvent ratio of 1:18 (w/v). The mixture was then extracted at different temperatures (50°C–90°C) and extraction times (45–90 min). The pH was adjusted from 3.5 to 6.5 using a buffer solution of 0.1 M citric acid and 0.1 M sodium citrate. After extraction, the liquid extract was filtered under vacuum using Whatman No. 4 filter paper. The filtrate was concentrated under reduced pressure at 50°C to obtain a viscous extract (approximately 25% moisture), and then stored in dark glass vials at 4°C until further analysis.

#### 2.3.2 The Screening Experiments

Single‐factor experiments were first used to preliminarily select conditions for extraction temperature, extraction time, pH, sonication time, and ultrasonic amplitude. The screening experiment was conducted to identify the factors affecting TPC and to exclude variables with negligible effects. A Plackett–Burman design (PBD) was used to screen the significance of the selected experimental variables. In the screening, five factors, including ultrasonic amplitude (Amp), sonication time (Stime), pH, extraction temperature (Temp), and extraction time (Time), were evaluated for their influence on TPC value. The screening matrix was generated as a fractional factorial design and analyzed using Minitab Statistical Software 22.

#### 2.3.3 Design of Experiment by Using Response Surface Methodology

Response surface methodology (RSM) was used to evaluate the linear, quadratic, and interactive effects of the selected variables on TPC. A central composite design (CCD) was applied within the RSM framework to optimize the significant variables. Optimization and statistical analysis were performed using Minitab Statistical Software 22. This approach allows the interaction among variables to be evaluated while reducing the number of experimental runs. The regression equations are represented as follows:

$$\begin{array}{c} Y=b_{o}+b_{1}.X_{1}+b_{2}.X_{2}+b_{3}.X_{3}+b_{11}.X_{12}+b_{22}.X_{22}\\ +b_{33}.X_{32}+b_{12}.X_{1}.X_{2}+b_{13}.X_{1}.X_{3}+b_{23}.X_{2}.X_{3} \end{array}$$

Variables X_1_, X_2_, X_3_, X_4_ are coded variables so these variables need to be converted to actual variables according to the following formula:

$$X_{i}=\frac{U_{i}-{U_{i}}^{0}}{∆U_{i}}\;\left(i=1,2,3,\ldots ,k\right)$$

where

$$∆U_{i}=\frac{{U_{i}}^{\max}-{U_{i}}^{\min}}{2};{U_{i}}^{0}=\frac{{U_{i}}^{\max}+{U_{i}}^{\min}}{2}$$

$U_{i}$: Actual value of factors/variables.

$X_{i}$: Encoding values of factors/variables.

${U_{i}}^{0}$: The actual value of variables at the center point.

#### 2.3.4 Production of Gummy Candy

The gummy candies were made with gelatin (250 bloom) soaked in water for 10 min to hydrate. Sugar, malt syrup, and water were mixed and boiled to 80°C with stirring. The hydrated gelatin was then mixed and heated to 80°C for 10 min with continuous stirring. Concentrated ELC extract was added at 70°C in concentrations of 0.5%, 1.0%, 1.5%, and 2.0%. The liquid mixture was poured into molds, allowed to cool, and refrigerated at 4°C for 24 h. The gummy candies were vacuum‐packed in plastic bags and stored at room temperature until the following analysis.

#### 2.3.5 Analytical Methods

##### 2.3.5.1 Determination of Total Phenolic Compound and CGA by UV–Vis Spectrophotometric Method

The total phenolic content was quantified using the Folin–Ciocalteu method (Agbor et al. 2014). The mixture was incubated for 60 min in the dark at room temperature. The absorbance was then determined at 765 nm using a UV–Vis spectrophotometer. The results were presented as milligrams gallic acid equivalent per gram dry weight (mg GAE/g DW).

Chlorogenic acid content was determined using a UV–Vis spectrophotometric method as described by Belay and Gholap (Belay and Gholap 2009). The absorbance of the extract was measured at 330 nm using a JASCO 770 UV–Vis spectrophotometer (JASCO, Japan).

##### 2.3.5.2 Determination of Total Phenolic Compound by Ultra High‐Performance Liquid Chromatography Analysis (UPLC)

Ten milligrams of graphitized carbon were mixed with 1 mL of the extract in a vial. The mixture was then filtered through a 0.45 μm nylon membrane filter prior to UPLC injection. TPC was quantified using a Thermo Ultimate 3000 UPLC system (Waltham, MA, USA) equipped with a C18 column (250 × 4.6 mm, 5 μm) and a UV detector set at 265 nm. The mobile phase consisted of (A) methanol and (B) Milli‐Q water acidified with 0.1% H₃PO₄. Separation was performed at a flow rate of 0.7 mL/min using the following gradient program: 97% B (0–0.5 min), 97%–83% B (0.5–8.0 min), 83%–70% B (8.0–10.0 min), 70%–55% B (10.0–15.0 min), 55%–5% B (15–20 min), 5%–97% B (20–22 min), and 97% B (22–23 min). The injection volume was 5 μL, and all analyses were conducted in triplicate (Vu et al., 2025).

##### 2.3.5.3 Determination of Antioxidant Activity by the DPPH Method

The 2,2‐diphenyl‐1‐picrylhydrazyl (DPPH) radical scavenging experiment was used to measure antioxidant activity, as described by Brand‐Williams (Brand‐Williams et al. 1995). Test tubes were filled with 0.1 mL of diluted extract and 3.9 mL of DPPH working solution. The mixture was vortexed for 20 s before incubating in the dark at room temperature for 60 min. The absorbance was measured at 517 nm. The percentage of DPPH radical scavenging activity was calculated using the following equation:

$$\mathrm{RSA}\;\left(\%\right)=\left[\left(A_{\text{contro}}l-A_{\text{sample}}\right)/A_{\text{control}}\right]\times 100$$

where A_control_ is the absorbance of the DPPH solution without sample, As_ample_ is the absorbance of the reaction mixture containing the extract.

A Trolox calibration curve was constructed using Trolox standard solutions at concentrations of 200–1000 μM. The calibration equation was:

$$y=0.0978x–2.8025,R^{2}=0.992$$

where y is the percentage of DPPH radical scavenging activity, x is the Trolox concentration (μM).

The antioxidant activity of the samples was then calculated from the Trolox calibration curve and expressed as μmol Trolox equivalents per gram of dry weight (μmol TE/g DW).

##### 2.3.5.4 Determination of Peroxide Value (PV)

The peroxide value was determined using the iodometric titration method described in ISO 3960‐2017, with minor modifications. Briefly, 30 g of gummy sample was melted at 60°C for 20 min and diluted with distilled water (1:4, w/v). Lipids were extracted using hexane, collected by phase separation, and the solvent was removed under vacuum evaporation at 45°C.

The recovered oil was dissolved in an acetic acid–isooctane mixture (3:2, v/v), followed by the addition of freshly prepared saturated potassium iodide solution. The liberated iodine was titrated with 0.01 N sodium thiosulfate using starch as an indicator. Peroxide value was expressed as meq O_2_/kg oil.

##### 2.3.5.5 Determination of Thiobarbituric Acid Reactive Substances (TBARS) Value

TBARS was determined according to the method of Buege and Aust (1978) with minor modifications. Gummy samples (30 g) were homogenized with 10% trichloroacetic acid (TCA) and centrifuged at 3600 rpm for 10 min (Buege and Aust 1978). An aliquot of supernatant was mixed with 0.02 M thiobarbituric acid (TBA) at a 1:1 ratio and heated in a boiling water bath for 20 min. After rapid cooling, absorbance was measured at 532 nm. TBARS content was expressed as mg malondialdehyde (MDA)/kg sample, quantified using a calibration curve prepared with 1,1,3,3‐tetramethoxypropane (TMP) as the MDA precursor.

##### 2.3.5.6 Determination of TPC, CGA and Antioxidant Activity in Gummy Candy

For the gummy candy samples, 1.0 g of each sample was cut into small pieces and mixed with 9 mL of distilled water. The mixture was homogenized until the gummy matrix was completely dispersed and then centrifuged at 3000 rpm. The clear supernatant was collected and used for the analysis of TPC, CGA, and antioxidant activity. The analytical procedures were the same as those used for the ELC extracts.

##### 2.3.5.7 Scanning Electron Microscopy Analysis

The surface morphology of untreated and ultrasound‐assisted extraction (UAE)‐treated ELC leaf tissues was examined using scanning electron microscopy (SEM). After extraction, the residual leaf tissue was collected and dried at low temperature until a stable moisture level was reached. The dried samples were then observed using a Phenom Pro Generation 5 scanning electron microscope (Phenom‐World, Netherlands). SEM micrographs were captured at selected magnifications (1000× and 10,000×) to compare the epidermal surface, cell wall integrity, pore formation, and tissue disruption between untreated and UAE‐treated samples.

##### 2.3.5.8 Statistical Analysis

All experiments were carried out in triplicate. Statistical analyses were conducted using Minitab version 22. A one‐way analysis of variance (ANOVA) was used to determine significant differences between treatments, and Tukey's multiple comparison test was used to identify differences between means. *p* < 0.05 indicated statistical significance.

## 3 Results and Discussion

### 3.1 Chemical Composition of ELC Leaves

Based on the chemical composition of the initial ELC leaf material, the leaves had a high moisture content of 83.7%. This indicates that pretreatment and drying are important steps for stabilizing the raw material and minimizing the loss of bioactive compounds during storage and extraction. The results also show that the main nutritional components, calculated on a dry weight basis, were relatively low, including protein at 4.3%, carbohydrate at 4.9%, total fiber at 1.7%, lipid at 1.2%, and total ash at 1.6%. Therefore, ELC leaves should not be considered a major source of energy or macronutrients. Instead, their value mainly lies in their bioactive compounds.

A notable finding is that the leaves contained a total phenolic content of 35.4 mg GAE/g DW and a chlorogenic acid content of 10.93 mg/g DW, together with high antioxidant activity of 395.8 μmol TE/g DW. These results provide an important scientific basis for selecting ELC leaves as the main material in this study, from pretreatment and extraction to the evaluation of biological activity and potential practical applications.

### 3.2 Effect of Ultrasonic Amplitude

**FIGURE 1:**
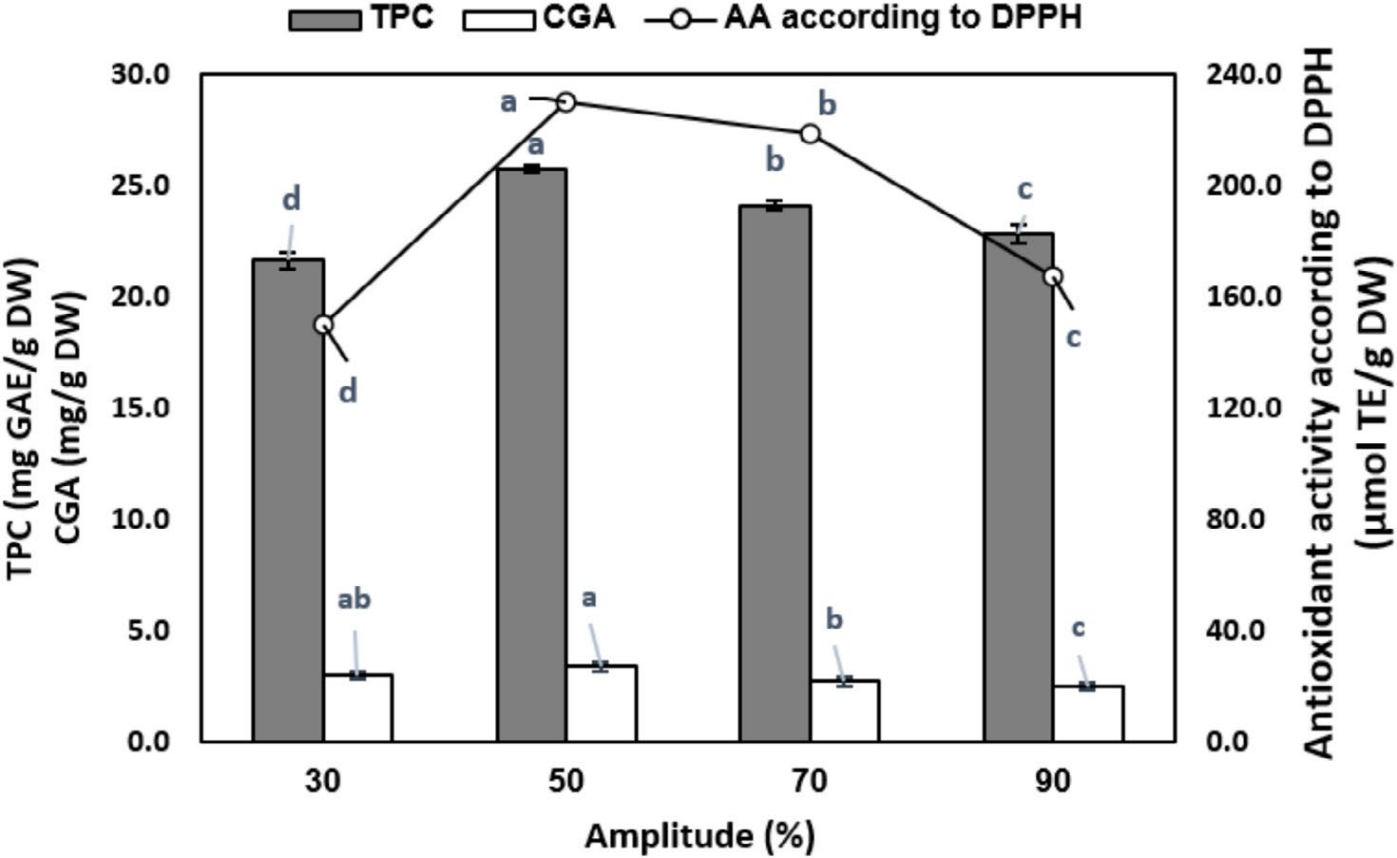
Effect of amplitude on TPC, CGA and AA according to DPPH of ELC extract. Sonication time: 5 min, amplitude: 30%–90%. Extraction conditions: Temperature: 70°C, time: 60 min, material‐to‐solvent: 1/18, ethanol concentration: 60%, pH: 5.5. The data represent mean values. The results from individual letters are statistically significant (*p* < 0.05).

As shown in Figure 1, increasing the amplitude from 30% to 50% significantly improved TPC, CGA, and antioxidant activity (*p* < 0.05). The highest values of TPC, CGA, and AA at 50% were 25.75 ± 0.15 mg GAE/g DW, 3.37 ± 0.25 mg/g DW, and 229.92 ± 1.68 μmol TE/g DW, respectively. When the amplitudes exceeded 70%, all parameters decreased.

Ultrasonic amplitude is a crucial factor influencing the extraction efficiency of bioactive compounds. The amplitude of ultrasonic waves directly affects cavitation intensity and the liberation of phenolic compounds by breaking cellular membranes and enhancing solvent infiltration. In addition, different ultrasonic amplitudes generated varying levels of heat within the extraction medium. This localized heating increased the temperature of the extract from approximately 45°C to75°C when the amplitude was raised from 30% to 90%. Such temperature elevation accelerates the thermal degradation of polyphenols, thereby reducing the overall extraction efficiency of these compounds (Chemat et al. 2017).

In some previous research, Borrás‐Enríquez et al. (2021) reported that mango pomace exhibited the highest TPC at 60% amplitude (Borrás‐Enríquez et al. 2021). Egüés et al. (2021) also noted the release of phenolic compounds from apple pomace at a moderate amplitude (50%), despite reduced DPPH activity at higher levels (Egüés et al. 2021). Similarly, Leksawasdi et al. (2022) found a 50% amplitude suitable for green bean hulls (Leksawasdi et al. 2022). Overall, a 50% amplitude was chosen as a suitable condition for further experiments.

### 3.3 Effect of Sonication Time

**FIGURE 2:**
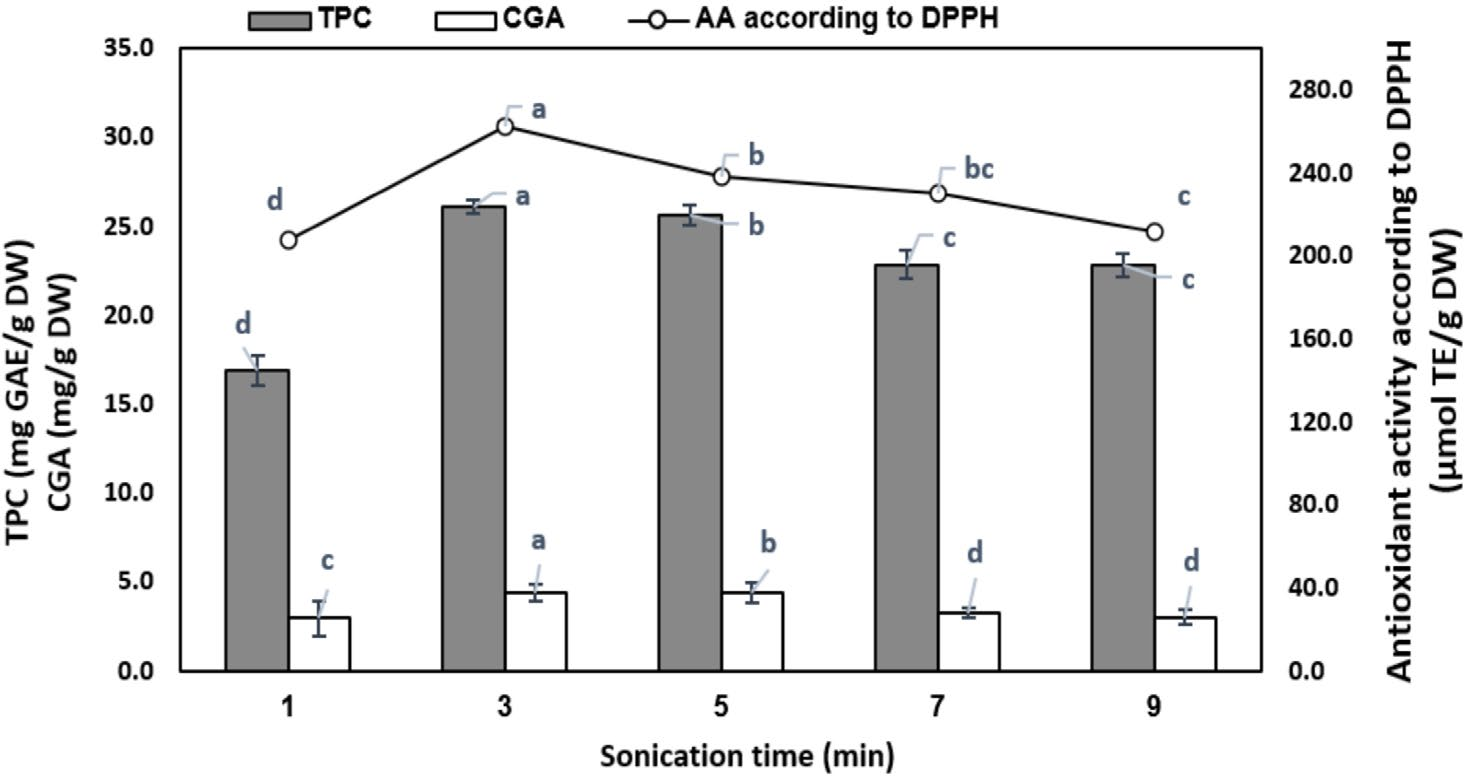
Effect of sonication time on TPC, CGA and AA of ELC extract. Sonication time: 1–9 min, amplitude: 50%. Extraction conditions: Temperature: 70°C, time: 60 min, material‐to‐solvent: 1/18, ethanol concentration: 60%, pH: 5.5. The data represent mean values. The results from individual letters are statistically significant (*p* < 0.05).

Figure 2 shows that a sonication time of 3 min yielded the highest extraction efficiency, with TPC, CGA, and AA reaching 26.13 ± 0.56 mg GAE/g DW, 4.40 ± 0.60 mg/g DW, and 262.64 ± 1.41 μmol TE/g DW, respectively. Extending the sonication time to 9 min resulted in a significant decline in all parameters (*p* < 0.05).

Short sonication enhances cell wall breakdown and solvent penetration, thereby increasing the release of phenolic compounds. A similar study in hog plum pulp found that extended sonication (30 min) resulted in a decrease in phenolic content (Ahmed et al. 2022).

Although the total temperature of the extract did not increase with longer sonication in this investigation, prolonged sonication time produces excessive hydroxyl radicals, which enhance phenolic oxidation and degradation (Shen et al. 2023). The localized micro‐hotspots formed during UAE may still cause thermal and oxidative stress to phenolic compounds (Chemat et al. 2017). These findings suggest that 3 min of sonication was a suitable time for the subsequent experiments.

### 3.4 Effect of pH

**FIGURE 3:**
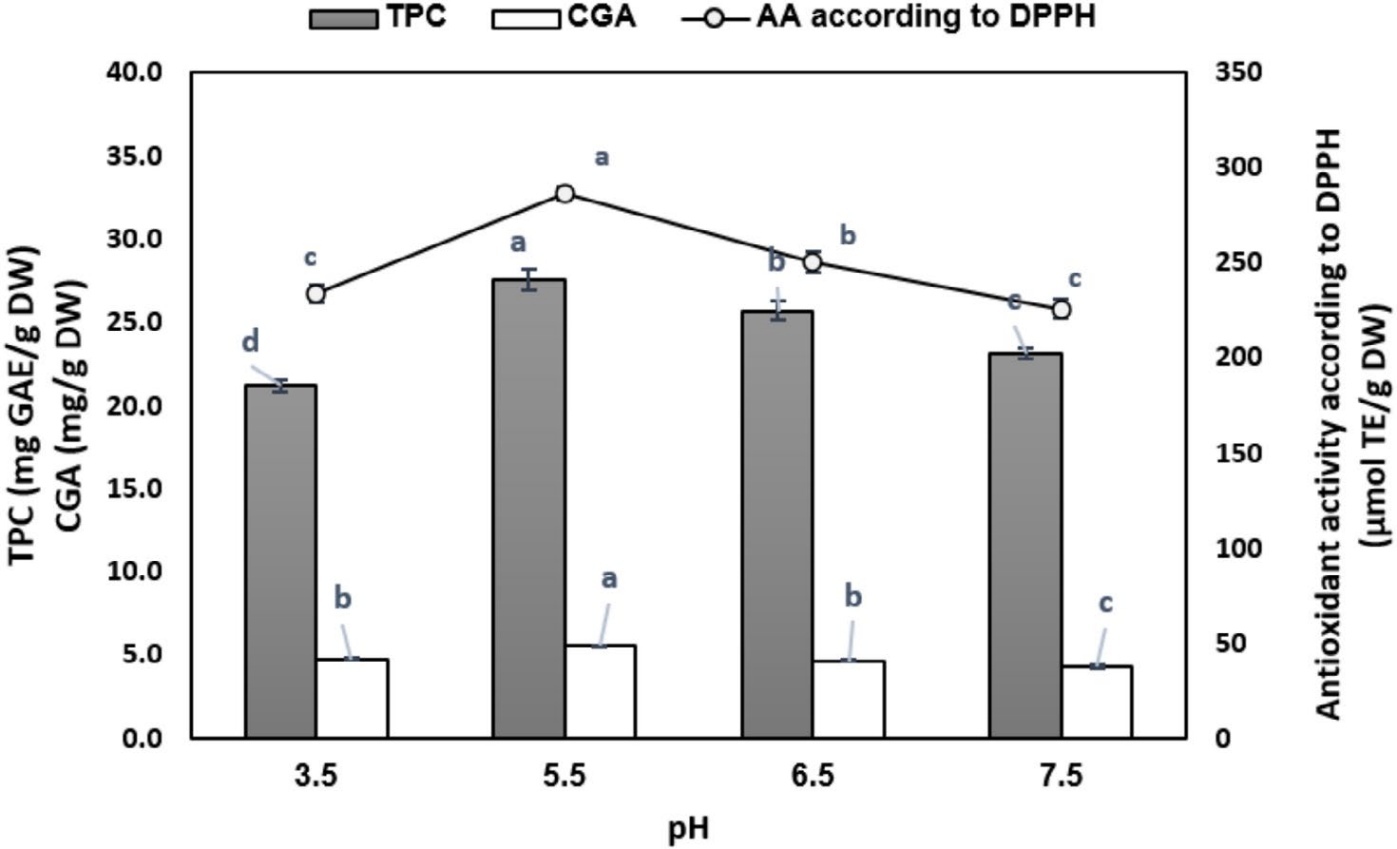
Effect of pH on TPC, CGA and AA of ELC extract. Sonication time: 3 min, amplitude: 50%. Extraction conditions: Temperature: 70°C, time: 60 min, material‐to‐solvent: 1/18, ethanol concentration: 60%, pH: 3.5–7.5. The data represent mean values. The results from individual letters are statistically significant (*p* < 0.05).

The results in Figure 3 show that pH significantly affects the extraction efficiency. TPC was highest at pH 5.5 with 27.56 ± 0.67 mg GAE/g DW, then decreased to 19.2 ± 0.3 mg GAE/g DW at pH 7.5. The CGA content and AA also reached the highest at pH 5.5, with 5.56 ± 0.15 mg/g DW and 286.65 ± 3.5 μmol TE/g DW, respectively. Friedman and Jürgens (2000) reported that some polyphenols are less stable at high pH, while chlorogenic acid is more stable in acidic and neutral environments, Mikucka et al. (2022) emphasized that weakly acidic–neutral pH helps maintain the stability and solubility of polyphenols, limiting oxidation during sonication (Friedman and Jürgens 2000; Mikucka et al. 2022). In the report of Gil‐Martín et al. (2022), the optimum pH and extraction conditions were crucial to maximize phenolic yield and preserve compound integrity (Gil‐Martín et al. 2022). Polyphenols, including chlorogenic acid, tend to remain stable in acidic to mildly neutral conditions, while higher pH levels make them much more susceptible to oxidation and breakdown (Grigorakis et al. 2020). Therefore, the extraction efficiency declined at pH 6.5 to 7.5, despite the same ultrasonic conditions. Overall, the pH 5.5 is the suitable condition for subsequent UAE experiments.

### 3.5 Effect of Extraction Temperature and Extraction Time

**FIGURE 4:**
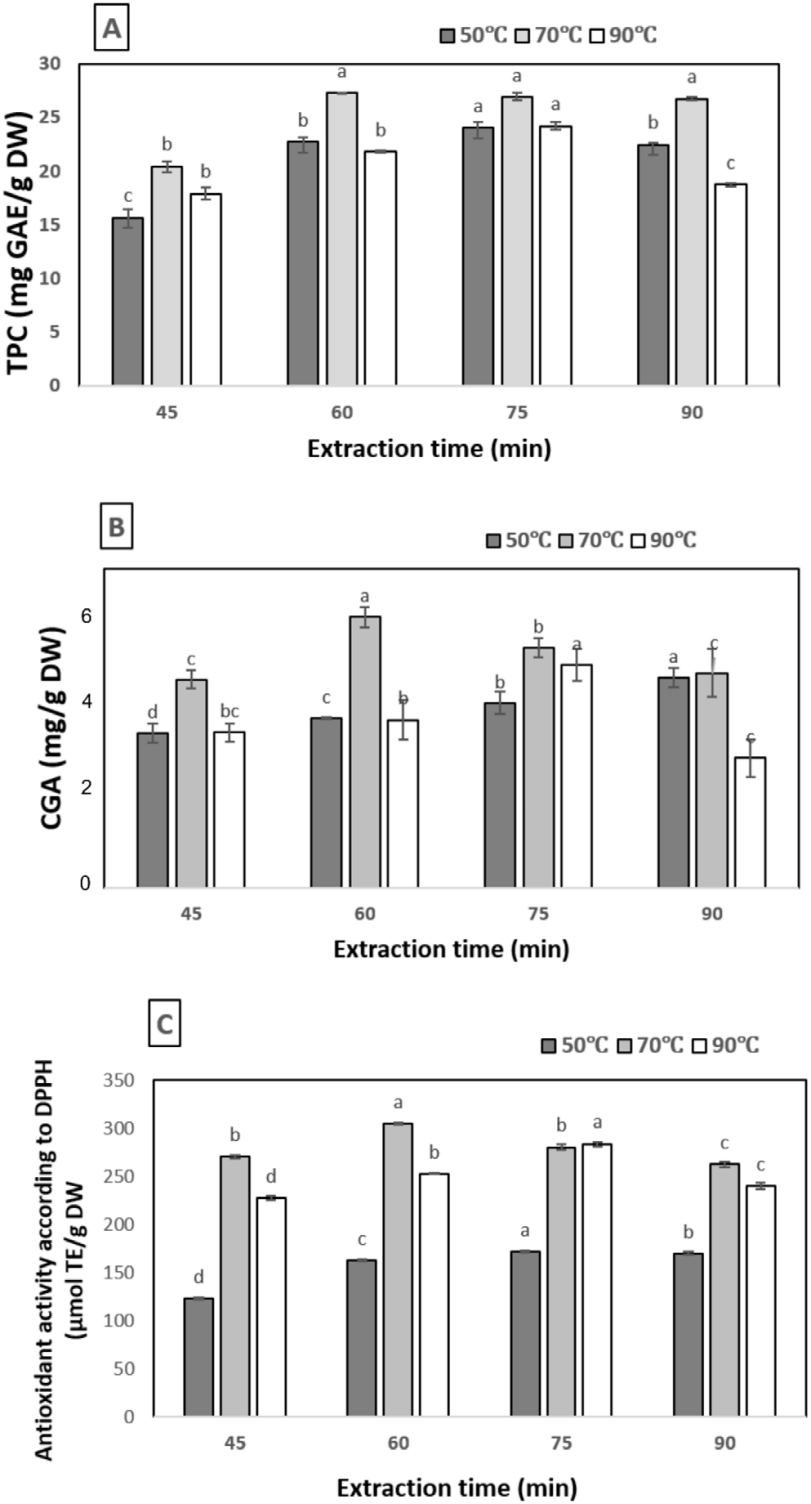
Effect of extraction temperature and extraction time on TPC, CGA and AA of ELC extract. Pretreatment conditions: Drying temperature 55°C, drying time: 6 h, sonication time: 3 min, amplitude: 50%. Extraction conditions: Temperature: 50°C–90°C, time: 45–90 min, material‐to‐solvent: 1/18, ethanol concentration: 60%, pH: 5.5. (A) Effect of temperature and time of extraction on the TPC; (B) Effect of temperature and time of extraction on the CGA content; (C) Effect of temperature and time of extraction on the antioxidant activity according to DPPH. The data represent mean values. The results from individual letters are statistically significant (*p* < 0.05).

The results in Figure 4 show that the extraction temperature and time after sonication have a significant impact on the TPC, CGA, and antioxidant activities in the ELC extract. The TPC, CGA, and AA were highest at an extraction temperature of 70°C for 60 min. In detail, TPC, CGA, and AA were 27.27 ± 0.07 mg GAE/g DW, 6.07 ± 0.18 mg/g DW, and 304.72 ± 0.83 μmol TE/g DW, respectively. However, when the extraction time and temperature were extended to 90 min at 90°C, a decline in bioactive compounds occurred.

As in some previous studies, ultrasonic waves help break the cell wall, increase the ability to penetrate the solvent and diffuse the active ingredient, but at too high a temperature and extraction time, they can destroy the structure of the plant cell (Chemat et al. 2017; Vilkhu et al. 2008). This phenomenon also corresponds to the principle that the interaction between suitable temperature and time creates a nonlinear relationship, with an optimal extreme point (Eriksson 2008; Myers and Montgomery 1996). Therefore, the condition of 70°C for 60 min was chosen as the suitable conditions for further application.

### 3.6 Determining the Influencing Variables for TPC

A Plackett–Burman design (PBD) was used to screen the critical UAE variables affecting TPC (Table 1). Five variables were investigated in 12 experimental runs. Twelve experimental runs with the variables' low (−) and high (+) levels yielded TPC values ranging from 14.76 to 28.09 mg GAE/g DW. The screening model showed a good fit, with R^2^ = 96.78%, adjusted R^2^ = 94.10%, and predicted R^2^ = 87.14% (Table 2). Temp, Time, and Stime significantly affected TPC (*p* < 0.05), whereas pH and amplitude were not significant (Table 2).

**TABLE 1:** Plackett–Burman screening design matrix.

Run order	Factors					TPC (mg GAE/g DW)
	Temp (°C)	Time (min)	pH	Stime (min)	Amp (%)	
1	60	50	6.5	4	70	16.16
2	80	70	3.5	4	70	28.09
3	80	70	3.5	4	30	27.88
4	60	50	3.5	4	70	15.88
5	60	50	3.5	2	30	14.76
6	80	50	6.5	2	30	16.50
7	80	50	3.5	2	70	16.65
8	60	70	6.5	2	70	18.28
9	60	70	6.5	4	30	22.67
10	80	70	6.5	2	70	23.44
11	60	70	3.5	2	30	17.66
12	80	50	6.5	4	30	22.22

**TABLE 2:** Coefficients list of the TPC response by model Plackett—Burman for the screening model of TPC.

Term	Coef	*F*‐value	*p*	Result
Constant	20.016	36.12	0.000	Significant
Temp	2.448	55.30	0.000	Significant
Etime	2.987	82.40	0.000	Significant
pH	−0.138	0.17	0.691	Not Significant
Stime	2.134	42.05	0.001	Significant
Amp	−0.266	0.65	0.450	Not Significant
*N* = 12	R^2^=	96.78%	S=	1.14008
	R^2^_adj._=	94.10%	R^2^ (pred)=	87.14%

Although pH 5.5 is suitable in single‐factor tests, pH (3.5–6.5) was not significant in the PBD (*p* > 0.05), suggesting a minor contribution to TPC relative to Temp, Time, and Stime.

The positive coefficients of Time, Temp, and Stime indicate that increasing these variables within the studied range increased TPC. Within the screening runs, the highest TPC (28.09 mg GAE/g DW) was obtained at 80°C, 70 min, and 4 min sonication. Therefore, extraction time, extraction temperature, and ultrasonic time were selected for further optimization.

This trend aligns with previous studies showing that TPC increases as extraction temperature, extraction time, and ultrasonic time reach optimal levels, but may level off or decline beyond those ranges (Altemimi et al. 2015; Antony and Farid 2022; Savic and Savic Gajic 2020).

### 3.7 Optimization for TPC by Using the Response Surface Method

Based on the PBD results, three significant variables were selected for CCD‐RSM optimization, and their coded levels are presented in Table 3. After screening, a central composite design (CCD) was applied in the RSM step, and the data were analyzed using Minitab Statistical Software 22 (Table 3). Twenty runs, including six center points, were performed to validate the CCD‐RSM model.

**TABLE 3:** Operating parameters for RSM‐CCD model.

Run order	Factors			TPC (mg GAE/g DW)
	Temp X_1_	Time X_2_	Stime X_3_	
1	80	70	4	28.16
2	80	70	4	27.01
3	80	70	4	27.75
4	80	70	5.68	23.34
5	75	80	5	25.75
6	85	60	3	25.39
7	85	80	5	20.15
8	80	70	2.32	26.11
9	85	60	5	23.63
10	80	86.82	4	25.02
11	80	70	4	27.70
12	75	80	3	27.13
13	85	80	3	22.11
14	75	60	5	26.08
15	88.41	70	4	20.62
16	80	53.18	4	26.94
17	80	70	4	27.77
18	80	70	4	27.59
19	75	60	3	28.09
20	71.59	70	4	27.57

The model had excellent goodness‐of‐fit (R^2^ = 98.75%, adjusted R^2^ = 97.63%, predicted R^2^ = 94.08%) and a low standard error (S = 0.38). ANOVA confirmed that the model was highly significant (*F‐value* = 87.85; *p* = 0.00). All three variables significantly affected TPC (*p* < 0.05), and the linear effects followed the order Temp, Stime, Time with *F‐values* of 378.32, 69.50, and 63.83, respectively. All quadratic terms were negative and significant (*p* < 0.05), indicating a curved response surface and the presence of an optimum region (Table 4). The strongest quadratic effect was observed for Temp^2^ (*F‐value* = 155.95) followed by Stime^2^ (*F‐value* = 105.56), and Time^2^ (*F‐value* = 34.40). Among the interaction terms, only Temp × Time was significant (*p* < 0.05), whereas Temp × Stime and Time × Stime were not (*p* > 0.05). The highest TPC values were observed near the center point (80°C, 70, 4 min), ranging from 27.01 to 28.16 mg GAE/g DW. The lack‐of‐fit was not significant (*p* = 0.465), confirming that the model adequately described the data.

**TABLE 4:** Analysis of variance and goodness‐of‐fit statistics and regression coefficients of the quadratic model for predicting TPC.

TPC (mg GAE/g DW)	Coeff. SC	*F*‐value	*p*	Result
Constant	27.663	87.85	0.000	Significant
X1	−2.011	378.32	0.000	Significant
X2	−0.826	63.83	0.000	Significant
X3	−0.862	69.50	0.000	Significant
X1*X1	−1.257	155.95	0.000	Significant
X2*X2	−0.590	34.40	0.000	Significant
X3*X3	−1.034	105.56	0.000	Significant
X1*X2	−0.684	25.63	0.000	Significant
X1*X3	−0.041	0.09	0.766	Not significant
X2*X3	0.054	0.16	0.699	Not significant
*N* = 20	R^2^=	98.75%	S=	0.382
	R^2^_adj._=	97.63%	Lack of fit=	0.465
	R^2^(pred)=	94.08%		

*Note:* Coeff. SC: Coefficient Standardized Coefficient.

**FIGURE 5:**
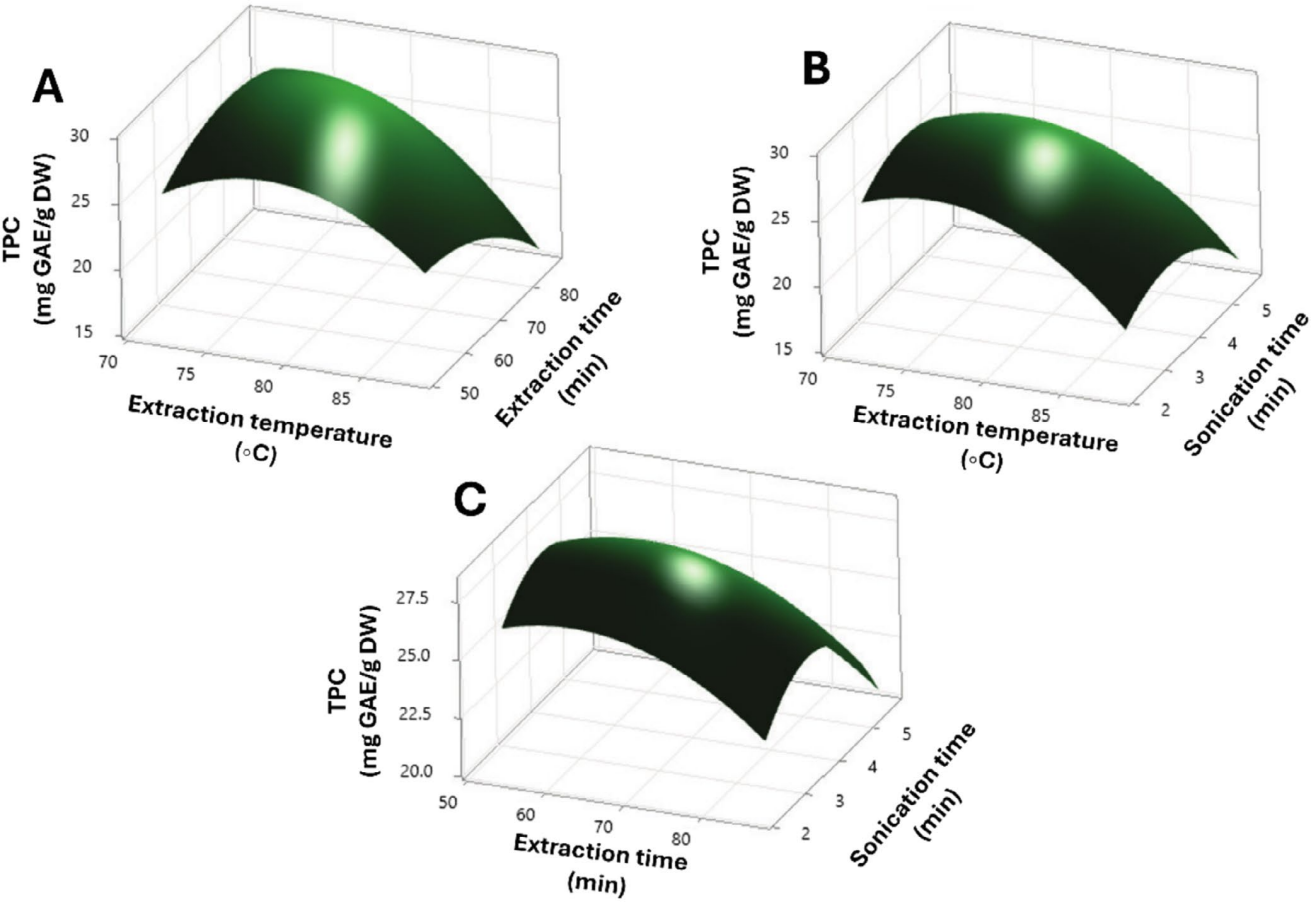
Response surface diagrams of TPC value using UAE. (A) Extraction temperature and extraction time interactive effect. (B) Extraction temperature and ultrasonic time interactive effect. (C) Extraction time and ultrasonic time interactive effect.

The response surface plots further supported the ANOVA results (Figure 5). The Temp × Time plot showed a clear curved surface, indicating that the simultaneous adjustment of extraction temperature and extraction time played an important role in TPC recovery (Figure 5A). Meanwhile, the Temp × Stime and Time × Stime plots showed smoother surfaces with less pronounced interaction patterns, consistent with the non‐significant interaction effects observed in the ANOVA (Figure 5B,C).

#### 3.7.1 The Predicted and Actual Value of TPC Under Optimal Conditions

Response optimizer analysis predicted the optimum conditions as 76.52°C, 66.82 min, and 3.58 min of sonication, with a predicted TPC of 28.67 mg GAE/g DW. The verification experiment was performed at 60% ethanol, pH 5.5, a material‐to‐solvent ratio of 1:18, 3.5 min sonication, 77°C, and 67 min extraction time. As shown in Table 5, the experimental TPC was in close agreement with the predicted value. These results further support the model's predictive accuracy.

**TABLE 5:** The TPC value of optimal parameters from regression equation.

	Units	Optimized by minitab 22	Actual value
Temp	°C	76.52	77
Time	min	66.82	67
Stime	min	3.58	3.6
TPC	mg GAE/dw	28.67	28.45 ± 0.5

### 3.8 Identification of Phenolic Compounds Using Ultra High‐Performance Liquid Chromatography (UPLC)

**FIGURE 6:**
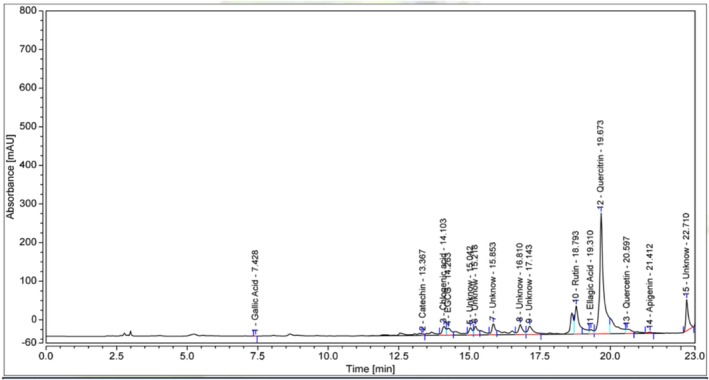
The phenolic profiles were extracted from ELC leaves at a wavelength of 265 nm by using UPLC‐UV analysis.

UPLC analysis was used to identify the phenolic compounds in the ELC leaves extract at the optimum extraction conditions. The chromatographic profile recorded at 265 nm is shown in Figure 6. Nine phenolic compounds were identified (Table 6), including apigenin, rutin, ellagic acid, quercitrin, quercetin, gallic acid, catechin, chlorogenic acid, and epigallocatechin gallate (EGCG).

**TABLE 6:** Quantitative composition (μg/mg) of phenolic compounds of ELC leaves extract.

Peak	Compound	Content (μg/mg)	Retention time (min)
1	Gallic acid	0.1 ± 0.1	7.428
2	Catechin	1.0 ± 0.1	13.367
3	Chlorogenic acid	5.6 ± 0.1	14.103
4	EGCG	2.9 ± 0.1	14.263
5	Rutin	6.2 ± 0.1	18.793
6	Ellagic acid	0.2 ± 0.1	19.310
7	Quercitrin	17.0 ± 0.2	19.673
8	Quercetin	0.9 ± 0.1	20.597
9	Apigenin	0.1 ± 0.1	21.412

The quantitative results indicate that quercitrin (17 ± 0.1 μg/mg) and rutin (6.2 ± 0.1 μg/mg) were the most abundant compounds, followed by chlorogenic acid (5.6 ± 0.1 μg/mg) and EGCG (2.9 ± 0.1 μg/mg). In contrast, gallic acid, ellagic acid, quercetin, and apigenin were detected at relatively low levels (< 1 μg/mg). The predominance of quercitrin and rutin is consistent with previous reports on Lamiaceae species, in which these flavonoids were identified as major bioactive constituents (Pudziuvelyte et al. 2020). Likewise, the presence of chlorogenic acid and catechin is consistent with reports for other Lamiaceae plants, including basil ( *Ocimum basilicum* ) (Teofilović et al. 2021). Overall, these findings suggest that ELC leaves are a promising source of flavonoids and phenolic acids, with potential application as functional ingredients.

### 3.9 Microstructure Analysis of *Elsholtzia ciliata* (Thunb) Hyland Leaf Surface

**FIGURE 7:**
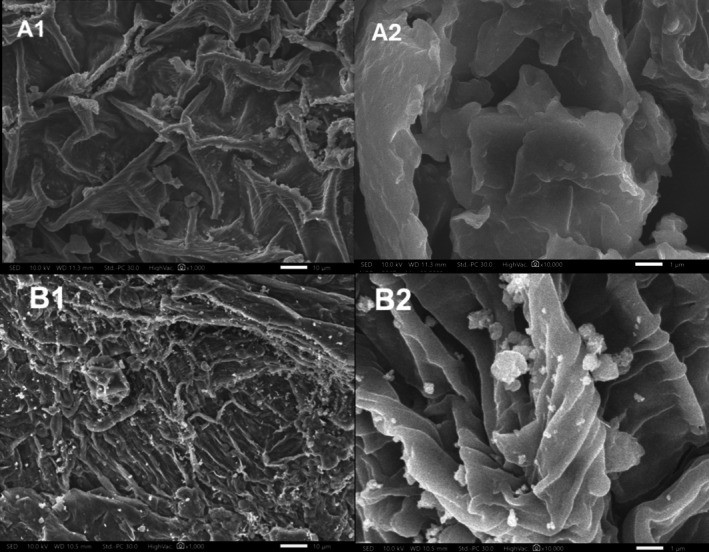
SEM micrographs of *Elsholtzia ciliata* (Thunb.) Hyland leaf surface at magnification of 1000× and 10,000× (A1, A2) untreated sample (B1, B2) UAE treated sample.

The SEM micrographs (Figure 7) clearly show structural changes between untreated and UAE‐treated leaf tissues. In the untreated samples (A1, A2), the epidermal surface is reasonably intact, with closely packed cell layers and unbroken cuticular structures. In contrast, the UAE‐treated samples (B1, B2) exhibit extensive surface disruption. Cavitation‐induced mechanical shear caused significant cell wall rupture, epidermal layer fragmentation, and the creation of fissures and enlarged pores. These morphological alterations are characteristic of ultrasonic cavitation, in which microjets and shockwaves disrupt the cellulose network and enhance solvent accessibility. The collapsed tissue structures further indicate increased release and diffusion of intracellular constituents (Shen et al. 2023).

Overall, the considerable damage to the structure shown in UAE‐treated samples illustrates the efficacy of ultrasound in facilitating the extraction of polyphenols and chlorogenic acid from *E. ciliata* leaves.

### 3.10 Evaluate the Quality of Gummy Candy According to Storage Time

**FIGURE 8:**
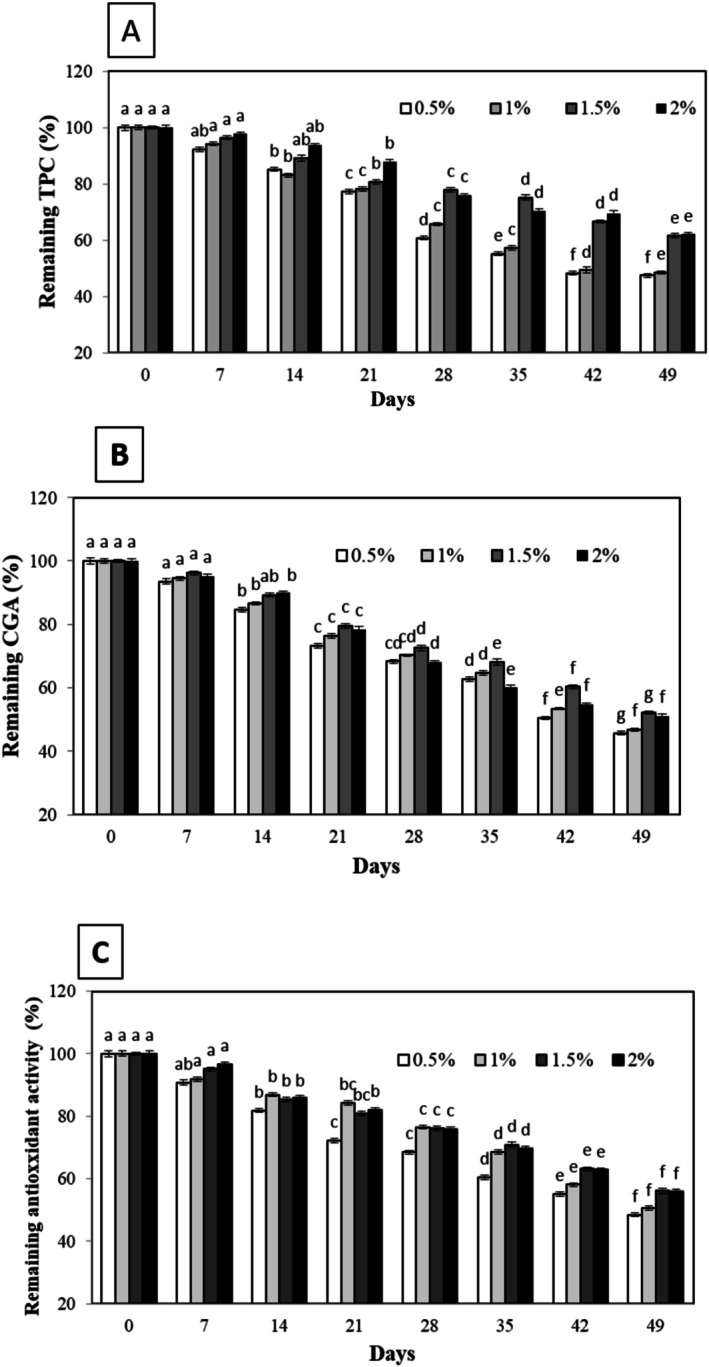
Changes in TPC, CGA content, and antioxidant activity of gummy samples supplemented with different levels of *Elsholtzia ciliata ext*ract during 49 days of storage. (A) Changes in TPC in gummy samples. (B) Changes in CGA content in gummy samples. (C) Changes in AA in gummy samples. The data presents mean values. At *p* < 0.05, the values from the various letters indicate statistical significance.

The stability of CGA, TPC, and antioxidant activity in gummy candies was monitored for 49 days under room temperature storage conditions. As shown in Figure 8, a gradual decrease in CGA, TPC, and AA content was observed at ELC extract concentrations of 0.5%, 1.0%, 1.5%, 2.0%, and the control sample with 0.0% concentrated extract. Furthermore, this decrease clearly slowed down as the ELC extract concentration increased. Notably, gummy candies containing 1.5% ELC extract maintained the highest CGA levels throughout the storage period (Figure 8B), providing the best balance between retaining high TPC and CGA content and sustained antioxidant activity.

This improved stability is primarily due to the antioxidant effects of ELC polyphenols, which actively scavenge free radicals and disrupt oxidation chain reactions. At the same time, the semi‐solid structure of the gelatin matrix restricts molecular mobility. It reduces oxygen diffusion, creating a less favorable environment for oxidation than liquid systems. A similar protective effect of structured food matrices on phenolic stability has been widely reported in previous studies (Demircan et al. 2024).

### 3.11 Peroxide Value (PV) and *Thiobarbituric Acid Reactive Substances* Value (TBARS)

**FIGURE 9:**
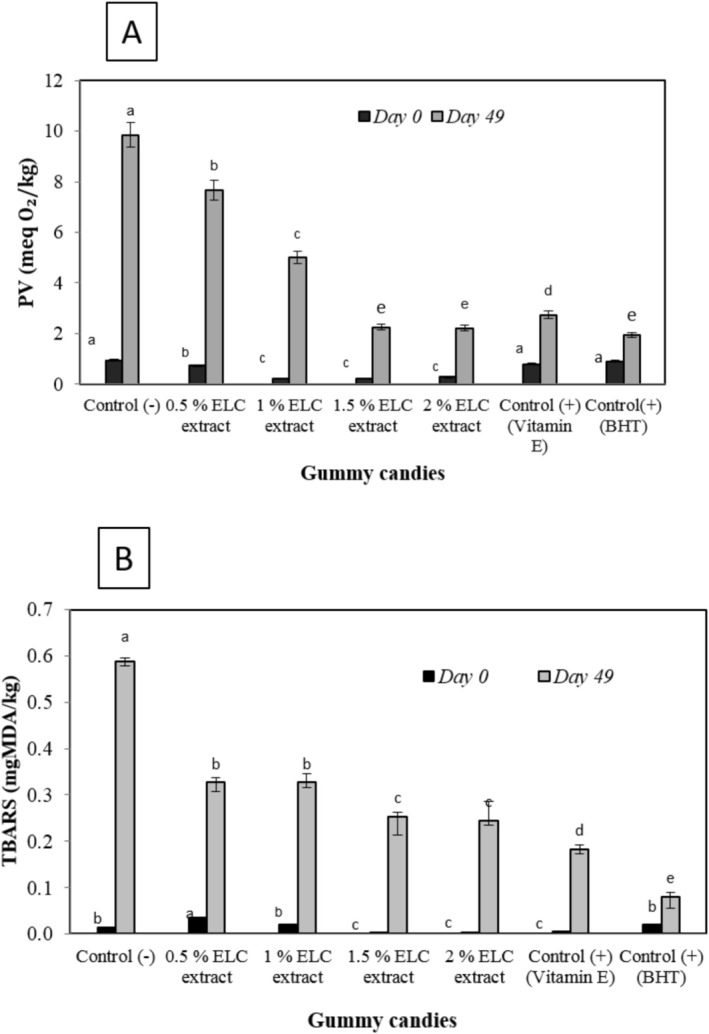
PV and TBARS at Day 0 and Day 49 in gummy candies enriched with *Elsholtzia ciliata ext*ract.

Figure 9 illustrates the change in PV and TBARS in gummy candies supplemented with ELC extract at different concentrations after 0 and 49 days of storage. At the start of storage, all gummy candy samples had similar PV levels, indicating that lipid oxidation had only just begun. However, as 49 days passed, the samples became increasingly different. The negative control sample oxidized rapidly, reaching a PV value of 9.85 meq O_2_/kg, TBARS of 0.60 mg MDA/kg, showing rapid lipid oxidation in the absence of a protective agent.

Conversely, the candy containing ELC extract oxidized significantly more slowly. Specifically, the higher the extract content, the greater the protective effect. The 1.5% formulation showed the best antioxidant effect, at PV only 2.25 meq O_2_/kg and TBARS was approximately 0.25 mg MDA/kg. This figure is comparable to vitamin E (2.74 meq O_2_/kg) and butylhydroxytoluene (BHT) (1.73 meq O_2_/kg). The significant reduction in PV in samples treated with ELC reflects the known behavior of phenolic compounds in lipid‐containing foods. Similar observations have been reported in meat when using phenolic‐rich plant extracts (Paglarini et al. 2023). The reduction in the accumulation of primary oxidation products in the gum samples may be due to the abundant phenolic compounds in the ELC extract, particularly rutin, quercitrin, and chlorogenic acid. These compounds effectively terminate the propagation phase of lipid oxidation, further reducing the formation of hydroperoxides.

Furthermore, the semi‐solid form of the gelatin matrix reduces oxygen diffusion, allowing phenolic compounds to achieve higher antioxidant efficacy compared to the liquid system (Strauss and Gibson 2004). Overall, the results show that ELC extract provides strong antioxidant protection, with a concentration of 1.5% providing preservative efficacy comparable to 100 ppm vitamin E and 100 ppm BHT.

Nevertheless, this study evaluated oxidative stability over time under a single room‐temperature condition using vacuum packaging. In real storage and distribution, oxidation kinetics can also be influenced by additional factors such as light exposure, temperature fluctuations, and oxygen availability. Therefore, these variables require further evaluation in future studies to better support industrial applicability.

## 4 Conclusion

Ultrasound‐assisted extraction was effective for the recovery of phenolic compounds from *Elsholtzia ciliata* leaves. The UPLC analysis showed that the extract was rich in quercitrin and rutin, chlorogenic acid, and EGCG.

When incorporated into fat‐containing gummy candies, the extract consistently slowed lipid oxidation during storage, as evidenced by reduced PV and TBARS levels. Among the tested formulations, 1.5% extract provided the strongest protection, performing on par with 100 ppm vitamin E and BHT. Overall, *E. ciliata* extract has potential as a natural, clean‐label antioxidant for improving the stability of fat‐containing confectionery products and other functional foods. Further studies should test more realistic storage conditions and evaluate sensory quality, texture stability, and scale‐up potential.

## Author Contributions

**Thi Anh Dao Dong:** conceptualization, investigation, methodology, validation, writing – review and editing, formal analysis, supervision, writing – original draft. **Nguyen Phuong Vi Truong:** conceptualization, investigation, writing – original draft, methodology, validation, software, formal analysis, data curation. **Tran Diem Ai Chau:** investigation, software, data curation. **Huu Hieu Nguyen:** conceptualization, investigation, methodology, formal analysis.

## Funding

The authors have nothing to report.

## Conflicts of Interest

The authors declare no conflicts of interest.

## Data Availability

The data that support the findings of this study are available from the corresponding author upon reasonable request.

## References

[fsn372080-bib-0001] Agbor, G. A. , J. A. Vinson , and P. E. Donnelly . 2014. “Folin‐Ciocalteau Reagent for Polyphenolic Assay.” International Journal of Food Science, Nutrition and Dietetics 3, no. 8: 147–156. 10.19070/2326-3350-1400028.

[fsn372080-bib-0002] Ahmed, T. , M. R. Rana , M. R. Maisha , A. S. M. Sayem , M. Rahman , and R. Ara . 2022. “Optimization of Ultrasound‐Assisted Extraction of Phenolic Content & Antioxidant Activity of Hog Plum ( *Spondias pinnata* L. f. Kurz) Pulp by Response Surface Methodology.” Heliyon 8, no. 10: e11109. 10.1016/j.heliyon.2022.e11109. 36281389 PMC9587330

[fsn372080-bib-0003] Altemimi, A. , R. Choudhary , D. G. Watson , and D. A. Lightfoot . 2015. “Effects of Ultrasonic Treatments on the Polyphenol and Antioxidant Content of Spinach Extracts.” Ultrasonics Sonochemistry 24: 247–255. 10.1016/j.ultsonch.2014.10.023. 25465093

[fsn372080-bib-0004] Antony, A. , and M. Farid . 2022. “Effect of Temperatures on Polyphenols During Extraction.” Applied Sciences 12, no. 4: 2107. 10.3390/app12042107.

[fsn372080-bib-0005] Belay, A. , and A. V. Gholap . 2009. “Characterization and Determination of Chlorogenic Acids (CGA) in Coffee Beans by UV‐Vis Spectroscopy.” African Journal of Pure and Applied Chemistry 3, no. 11: 234–240. http://www.academicjournals.org/ajpac.

[fsn372080-bib-0006] Borrás‐Enríquez, A. J. , E. Reyes‐Ventura , S. J. Villanueva‐Rodríguez , and L. Moreno‐Vilet . 2021. “Effect of Ultrasound‐Assisted Extraction Parameters on Total Polyphenols and Its Antioxidant Activity From Mango Residues ( *Mangifera indica* L. Var. Manililla).” Separations 8, no. 7: 94. 10.3390/separations8070094.

[fsn372080-bib-0007] Bouafia, M. , N. Colak , F. A. Ayaz , et al. 2021. “The Optimization of Ultrasound‐Assisted Extraction of Centaurea sp. Antioxidative Phenolic Compounds Using Response Surface Methodology.” Journal of Applied Research on Medicinal and Aromatic Plants 25: 100330. 10.1016/j.jarmap.2021.100330.

[fsn372080-bib-0008] Brand‐Williams, W. , M. E. Cuvelier , and C. Berset . 1995. “Use of a Free Radical Method to Evaluate Antioxidant Activity.” LWT ‐ Food Science and Technology 28: 25–30. 10.1016/S0023-6438(95)80008-5.

[fsn372080-bib-0009] Buege, J. A. , and S. D. Aust . 1978. “Microsomal Lipid Peroxidation.” In Methods in Enzymology, edited by S. Fleischer and L. Packer , vol. 52, 302–310. Academic Press. 10.1016/S0076-6879(78)52032-6. 672633

[fsn372080-bib-0010] Casterline, J. L. , C. J. Oles , and Y. Ku . 1999. “Measurement of Sugars and Starches in Foods by a Modification of the AOAC Total Dietary Fiber Method.” Journal of AOAC International 82, no. 3: 759–765. 10.1093/jaoac/82.3.759. 10367393

[fsn372080-bib-0011] Chemat, F. , N. Rombaut , A.‐G. Sicaire , A. Meullemiestre , A.‐S. Fabiano‐Tixier , and M. Abert‐Vian . 2017. “Ultrasound‐Assisted Extraction of Food and Natural Products: Mechanisms, Techniques, Combinations, Protocols and Applications.” Ultrasonics Sonochemistry 34: 540–560. 10.1016/j.ultsonch.2016.06.035. 27773280

[fsn372080-bib-0012] Demircan, H. , R. A. Oral , O. S. Toker , and I. Palabiyik . 2024. “Investigation of the Effects of Phenolic Extracts Obtained From Agro‐Industrial Food Wastes on Gelatin Modification.” ACS Omega 9, no. 18: 20263–20276. 10.1021/acsomega.4c00690. 38737019 PMC11080024

[fsn372080-bib-0013] Egüés, I. , F. Hernandez‐Ramos , I. Rivilla , and J. Labidi . 2021. “Optimization of Ultrasound Assisted Extraction of Bioactive Compounds From Apple Pomace.” Molecules 26, no. 13: 3783. 10.3390/molecules26133783. 34206325 PMC8270251

[fsn372080-bib-0014] Eriksson, L. 2008. Design of Experiments: Principles and Applications. MKS Umetrics AB.

[fsn372080-bib-0015] Friedman, M. , and H. S. Jürgens . 2000. “Effect of pH on the Stability of Plant Phenolic Compounds.” Journal of Agricultural and Food Chemistry 48, no. 6: 2101–2110. 10888506 10.1021/jf990489j

[fsn372080-bib-0016] Gil‐Martín, E. , T. Forbes‐Hernández , A. Romero , D. Cianciosi , F. Giampieri , and M. Battino . 2022. “Influence of the Extraction Method on the Recovery of Bioactive Phenolic Compounds From Food Industry By‐Products.” In Food Chemistry, vol. 378, 131918. Elsevier. 10.1016/j.foodchem.2021.131918. 35085901

[fsn372080-bib-0017] Grigorakis, S. , A. Halahlah , and D. P. Makris . 2020. “Batch Stirred‐Tank Green Extraction of *Salvia fruticosa* Mill. Polyphenols Using Newly Designed Citrate‐Based Deep Eutectic Solvents and Ultrasonication Pretreatment.” Applied Sciences (Switzerland) 10, no. 14: 4774. 10.3390/app10144774.

[fsn372080-bib-0018] Hames, B. , C. Scarlata , and A. Sluiter . 2008. “Determination of Protein Content in Biomass: Laboratory Analytical Procedure (LAP); Issue Date 05/23/2008.” https://www.nrel.gov.

[fsn372080-bib-0019] Leksawasdi, N. , S. Taesuwan , T. Prommajak , et al. 2022. “Ultrasonic Extraction of Bioactive Compounds From Green Soybean Pods and Application in Green Soybean Milk Antioxidants Fortification.” Food 11, no. 4: 588. 10.3390/foods11040588. PMC8871011 35206064

[fsn372080-bib-0020] Lohani, U. C. , and K. Muthukumarappan . 2021. “Study of Continuous Flow Ultrasonication to Improve Total Phenolic Content and Antioxidant Activity in Sorghum Flour and Its Comparison With Batch Ultrasonication.” Ultrasonics Sonochemistry 71: 105402. 33310455 10.1016/j.ultsonch.2020.105402 PMC7786600

[fsn372080-bib-0021] Martín‐García, B. , M. J. Aznar‐Ramos , V. Verardo , and A. M. Gómez‐Caravaca . 2022. “The Establishment of Ultrasound‐Assisted Extraction for the Recovery of Phenolic Compounds and Evaluation of Their Antioxidant Activity From *Morus alba* Leaves.” Food 11, no. 3: 314. 10.3390/foods11030314 PMC8834592 35159465

[fsn372080-bib-0022] Mikucka, W. , M. Zielinska , K. Bulkowska , and I. Witonska . 2022. “Recovery of Polyphenols From Distillery Stillage by Microwave‐Assisted, Ultrasound‐Assisted and Conventional Solid–Liquid Extraction.” Scientific Reports 12, no. 1: 3232. 35217709 10.1038/s41598-022-07322-0 PMC8881464

[fsn372080-bib-0023] Myers, R. H. , and D. C. Montgomery . 1996. Response Surface Methodology. Taylor & Francis.

[fsn372080-bib-0024] Paglarini, C. S. , V. A. S. Vidal , I. A. Neri‐Numa , G. M. Pastore , and M. A. R. Pollonio . 2023. “Effect of Commercial Plant Extracts on the Oxidative Stability of Mechanically Deboned Poultry Meat During Chilled Storage.” Food Research International 164: 112358. 10.1016/j.foodres.2022.112358. 36737946

[fsn372080-bib-0025] Pudziuvelyte, L. , V. Jakštas , L. Ivanauskas , et al. 2018. “Different Extraction Methods for Phenolic and Volatile Compounds Recovery From *Elsholtzia ciliata* Fresh and Dried Herbal Materials.” Industrial Crops and Products 120: 286–294. 10.1016/j.indcrop.2018.04.069.

[fsn372080-bib-0026] Pudziuvelyte, L. , M. Liaudanskas , A. Jekabsone , I. Sadauskiene , and J. Bernatoniene . 2020. “ *Elsholtzia ciliata* (Thunb.) Hyl. Extracts From Different Plant Parts: Phenolic Composition, Antioxidant, and Anti‐Inflammatory Activities.” Molecules 25, no. 5: 1153. 10.3390/molecules25051153. 32150805 PMC7179165

[fsn372080-bib-0027] Pudziuvelyte, L. , M. Stankevicius , A. Maruska , et al. 2017. “Chemical Composition and Anticancer Activity of *Elsholtzia ciliata* Essential Oils and Extracts Prepared by Different Methods.” Industrial Crops and Products 107: 90–96.

[fsn372080-bib-0028] Savic, I. M. , and I. M. Savic Gajic . 2020. “Optimization of Ultrasound‐Assisted Extraction of Polyphenols From Wheatgrass ( *Triticum aestivum* L.).” Journal of Food Science and Technology 57, no. 8: 2809–2818. 10.1007/s13197-020-04312-w. 32624589 PMC7316939

[fsn372080-bib-0029] Shen, L. , S. Pang , M. Zhong , et al. 2023. “A Comprehensive Review of Ultrasonic Assisted Extraction (UAE) for Bioactive Components: Principles, Advantages, Equipment, and Combined Technologies.” In Ultrasonics Sonochemistry, vol. 101, 106646. Elsevier B.V. 10.1016/j.ultsonch.2023.106646. 37862945 PMC10594638

[fsn372080-bib-0030] Sim, Y. Y. , W. T. J. Ong , and K. L. Nyam . 2019. “Effect of Various Solvents on the Pulsed Ultrasonic Assisted Extraction of Phenolic Compounds From *Hibiscus cannabinus* L. Leaves.” Industrial Crops and Products 140: 111708.

[fsn372080-bib-0031] Sluiter, A. , B. Hames , R. Ruiz , C. Scarlata , J. Sluiter , and D. Templeton . 2008. “Determination of Ash in Biomass: Laboratory Analytical Procedure (LAP).” https://www.nrel.gov.

[fsn372080-bib-0032] Strauss, G. , and S. M. Gibson . 2004. “Plant Phenolics as Cross‐Linkers of Gelatin Gels and Gelatin‐Based Coacervates for Use as Food Ingredients.” Food Hydrocolloids 18, no. 1: 81–89. 10.1016/S0268-005X(03)00045-6.

[fsn372080-bib-0033] Teofilović, B. , N. Grujić‐Letić , M. Karadžić , et al. 2021. “Analysis of Functional Ingredients and Composition of *Ocimum basilicum* .” South African Journal of Botany 141: 227–234. 10.1016/j.sajb.2021.04.035.

[fsn372080-bib-0034] Vilkhu, K. , R. Mawson , L. Simons , and D. Bates . 2008. “Applications and Opportunities for Ultrasound Assisted Extraction in the Food Industry—A Review.” Innovative Food Science & Emerging Technologies 9, no. 2: 161–169.

[fsn372080-bib-0035] Wang, X. , X. Gong , and B. Lin . 2024. “Optimization of Ultrasonic Pretreatment and Analysis of Chlorogenic Acid in Potato Leaves.” Scientific Reports 14, no. 1: 10613. 10.1038/s41598-024-61139-7. 38719831 PMC11079030

[fsn372080-bib-0036] Yusoff, I. M. , Z. Mat Taher , Z. Rahmat , and L. S. Chua . 2022. “A Review of Ultrasound‐Assisted Extraction for Plant Bioactive Compounds: Phenolics, Flavonoids, Thymols, Saponins and Proteins.” In Food Research International, vol. 157, 111268. Elsevier. 10.1016/j.foodres.2022.111268. 35761580

[fsn372080-bib-0037] Zeng, G. , Y. Ran , X. Huang , et al. 2022. “Optimization of Ultrasound‐Assisted Extraction of Chlorogenic Acid From Tobacco Waste.” International Journal of Environmental Research and Public Health 19, no. 3: 1555. 35162594 10.3390/ijerph19031555 PMC8835221

